# Supplementary material for: Global and regional ocean mass budget closure since 2003
Source: Nat Commun. 2024 Feb 15;15:1416. doi: 10.1038/s41467-024-45726-w (PMC10869725; doi:10.1038/s41467-024-45726-w)
Supplement: Supplementary file 1 — Supplementary Information [file 41467_2024_45726_MOESM1_ESM.pdf]

Global and regional ocean mass budget closure since 2003
Supplementary Figures and Tables

Supplementary Figure 1:

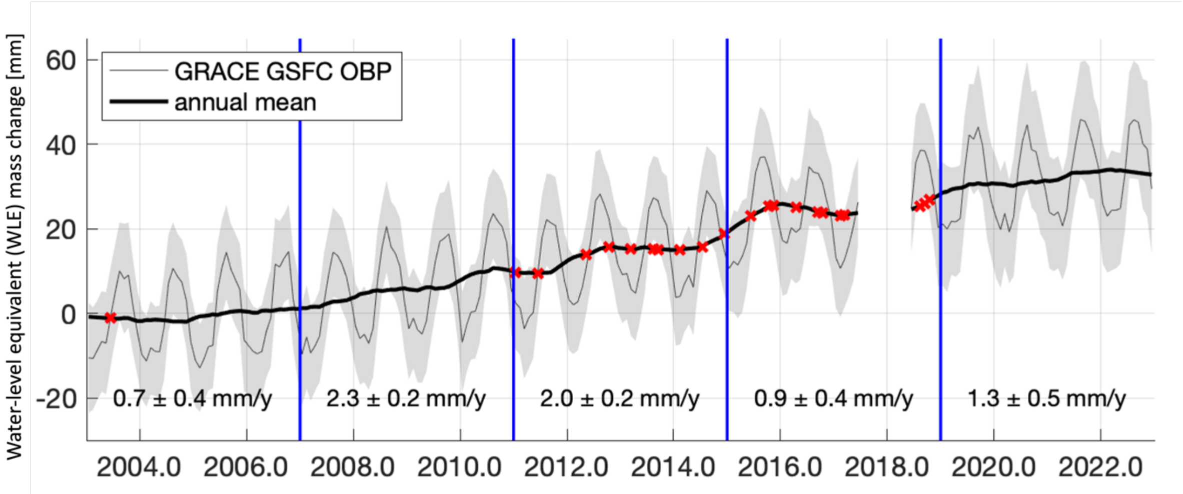

Supplementary Figure 1. ***GRACE-GFO global ocean mass timeseries****. GRACE-GFO global ocean mass timeseries*[16] *(in grey) and its corresponding moving annual mean (in bold, black). Trend estimates and 1σ uncertainty are given for five 4-year periods indicated by vertical blue lines. Months that are missing from the GRACE time series have been interpolated and are markered in red. The shaded grey areas represent the 1σ uncertainties.*

Supplementary Figure 2:

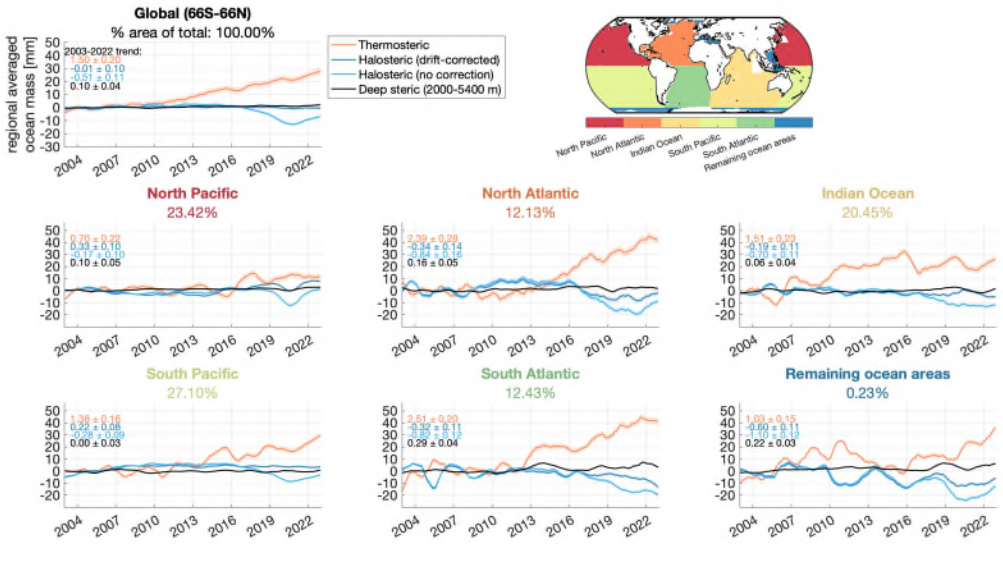

Supplementary Figure 2. ***Regional time series of steric sea level changes****. As Figure 3 in the main text, but for Thermosteric, Halosteric, Halosteric effects without drift correction (see Methods), and deep steric steric signal below 2000 meters).*

Supplementary Figure 3:

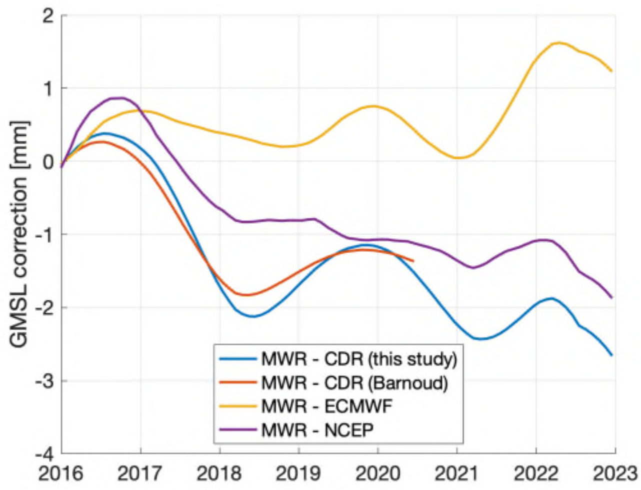

Supplementary Figure 3. ***Global-mean drift correction of Jason-3 Wet Troposphere Correction****. Global mean sea level correction [millimeters] from replacing the Microwave Radiometer (MWR) Wet Troposphere Correction (WTC) with two modeled WTCs, ECMWF,*

yellow, and NCEP, purple and two estimates from observed water vapor (Climate Data Records, CDR), from Barnoud et al, 2022 (red) and this study (blue).

Supplementary Figure 4:

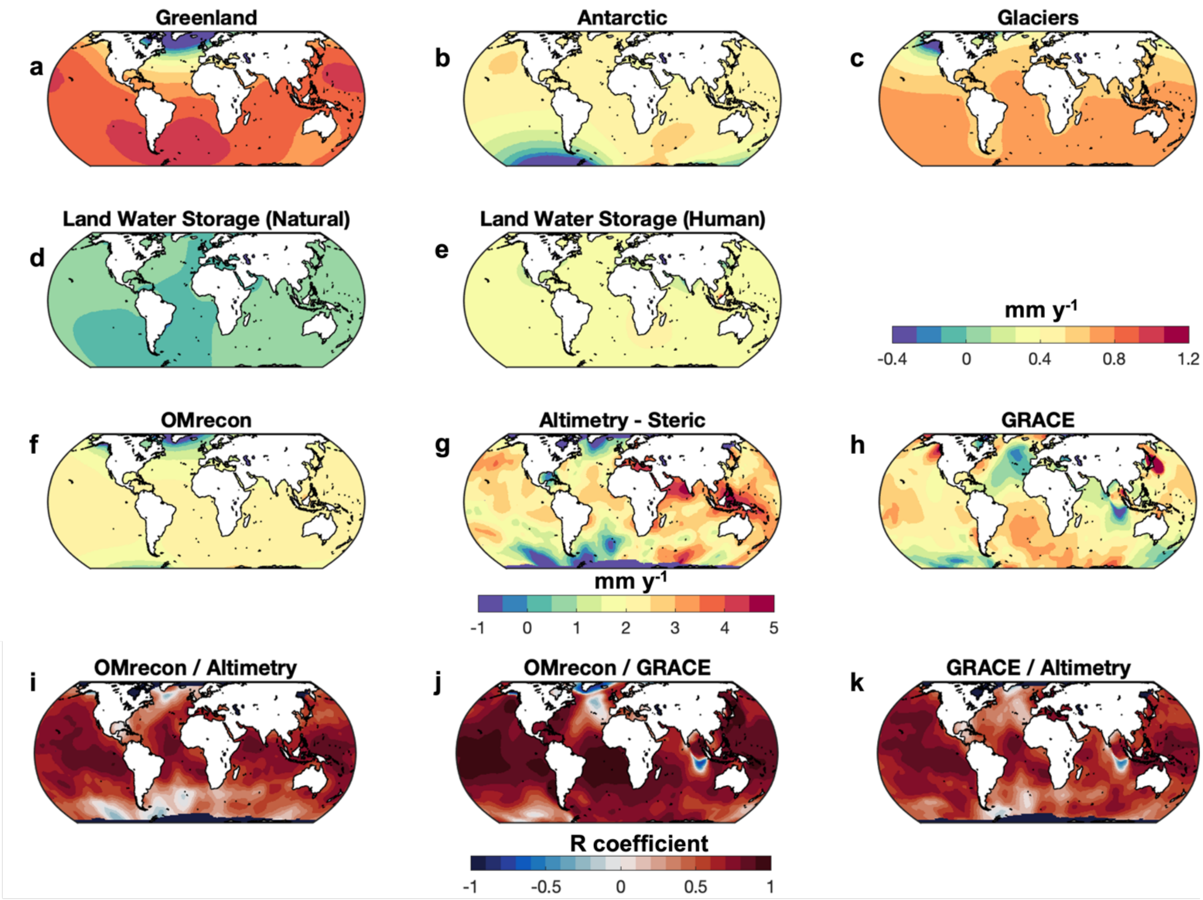

Supplementary Figure 4. ***Trend and correlation maps for 01/2003 – 12/2022 .a.*** *GRD-induced relative sea level change [mm* y^{-1}*] due to Greenland mass change, including peripheral glaciers.* ***b****.,* ***c., d.*** *and* ***e.*** *same as* ***a.*** *but for Antarctica, including peripheral glaciers, glaciers (excl. Greenland and Antarctica periphery), and natural land water storage and human-caused land water storage, respectively.* ***f****. the sum of* ***a-e*** *minus the atmospheric mass change (OMrecon).* ***g.*** *ocean mass change from steric-corrected Altimetry.* ***h.*** *GRACE observed mass change. All maps have been deseasonalized and a 500-km spatial filter has been applied to* ***g*** *and* ***h****.* ***i-k.*** *The correlation coefficient (R) between* ***f / g (i), f / h*** *(****j****), and* ***h / g*** *(****k****).*

Supplementary Figure 5:

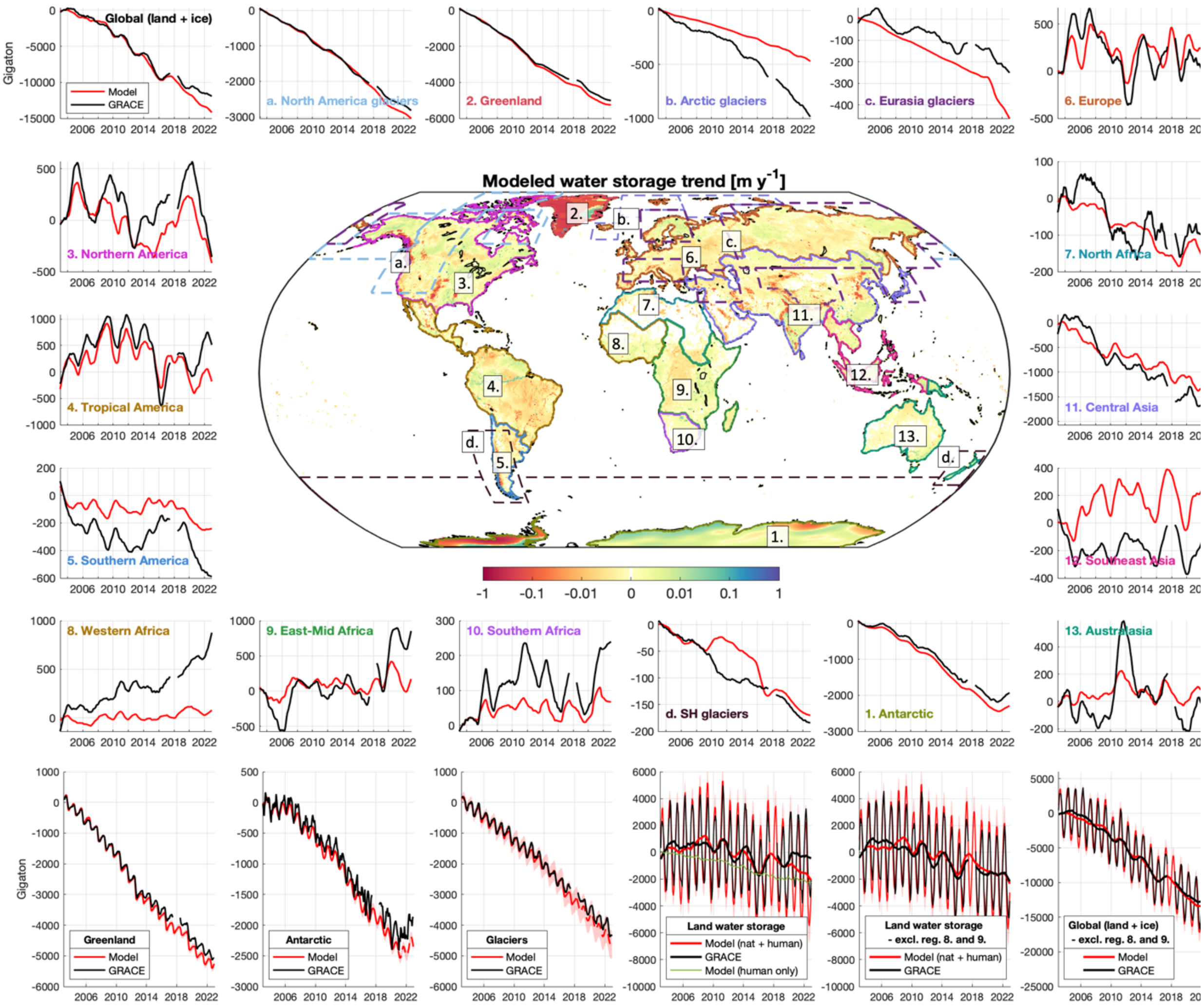

Supplementary Figure 5. ***Regional time series of land water storage****. Central map: Modeled water storage trends [m y^{-1}] from 2003-2022 used in this study to compute the GRD-induced ocean mass change for OMrecon. Small panels surrounding map: Regional 12-month averaged mass anomalies [Gigaton] are shown for both the modelled water storage (red) and land-mass change observed from GRACE (black). The title colors correspond to an outline on the central map. Boxes with dashed lines indicate regions with glacier mass. Boxes with solid line indicate ice sheets (including peripheral glaciers). Boxes with no outline indicates LWS (both natural and human caused LWS). LWS and glacial mass have been separated using the methodology described in methods. Bottom panels from left: Modelled (red) and GRACE-*

measured (black) monthly water mass anomalies for Greenland, Antarctic, glaciers, land water storage (total) and separately the human-caused LWS (green), LWS excluding region 8 and 9 (mid-Africa) and global water storage (ice + land) excluding region 8 and 9 (mid-Africa). For LWS and global, thick lines indicate the 12-month average.

Supplementary Figure 6:

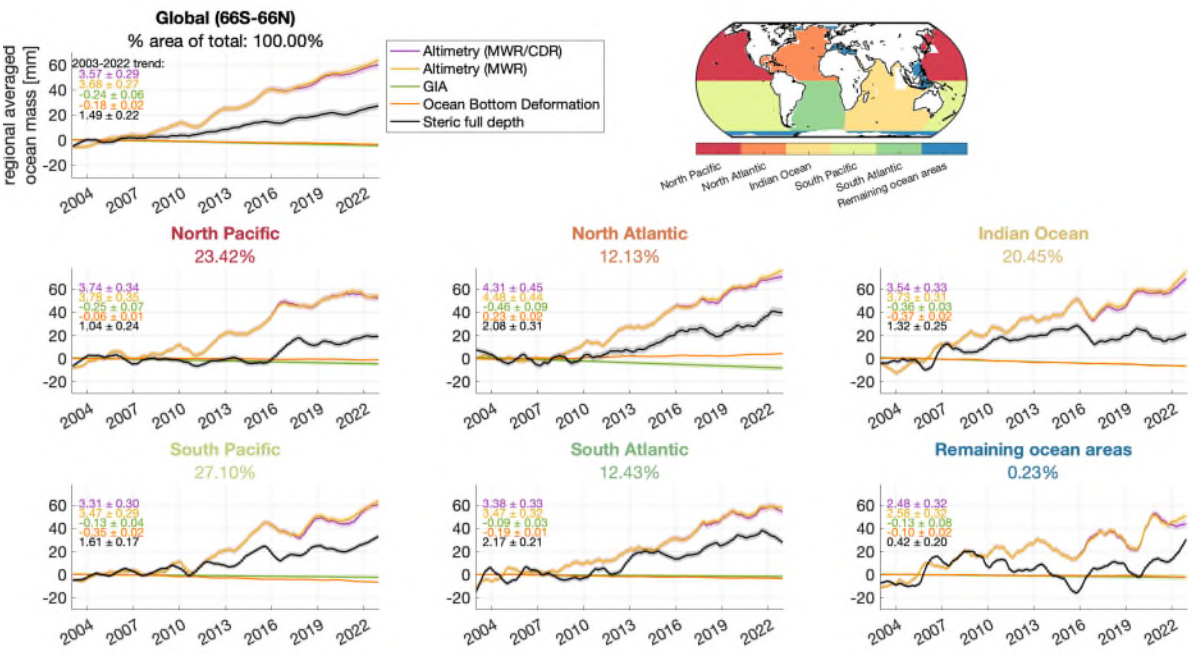

Supplementary Figure 6. ***Regional time series of altimetry-observed sea level, GIA, Ocean Bottom Deformation and Steric sea level.*** *As for Figure 3 in the main text, but for Altimetry measured sea level anomalies with the CDR WTC correction as adapted in this study, without the correction for steric, Glacial Isostatic Adjustment, and Ocean Bottom Deformation effects.*

Supplementary Figure 7:

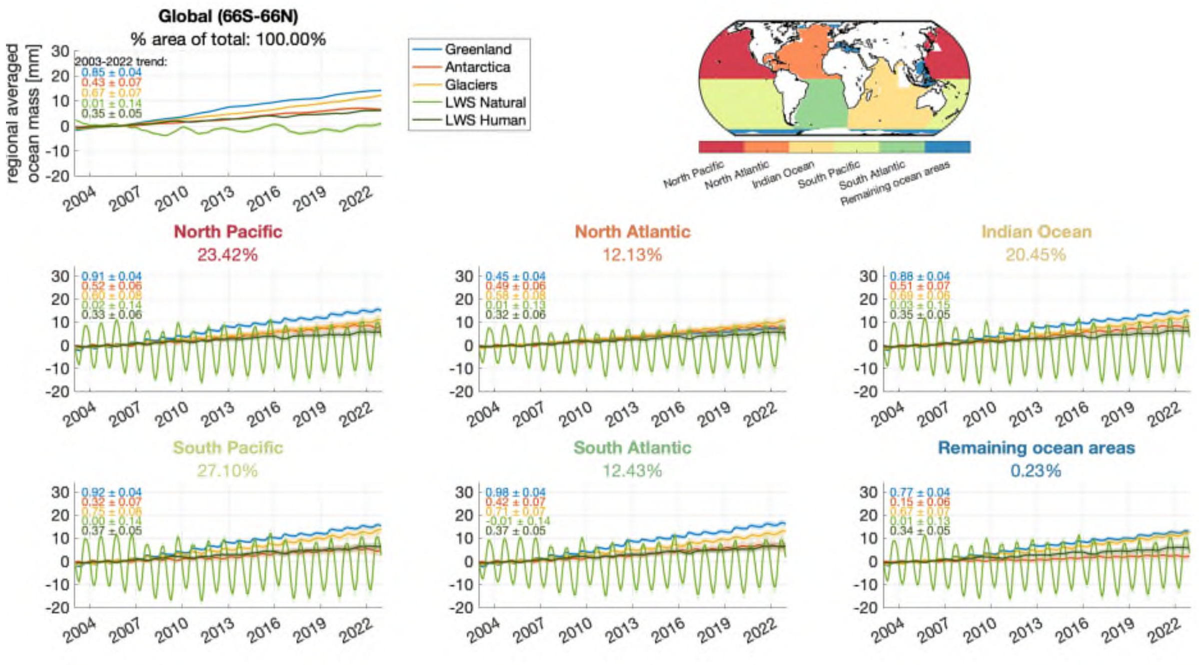

Supplementary Figure 7. ***Regional time series of the contributions to ocean mass change.*** *As Figure 3 in the main text, but for ocean mass change due to loading change in Greenland, Antarctica (both including peripheral glaciers), Glaciers (excl. Greenland and Antarctica), and natural and human-caused Land Water Storage (LWS). The regional figures are without any temporal averaging (i.e. monthly sampling).*

Supplementary Table 1. Components of the sea level budget for different ocean mask applied. *First column: Linear trends for the full ocean (except two areas related two the megathrust earthquake off the coast of in Sumatra and Japan) over the period 01/2003 to 12/2022. Second column: same as first column, but where additionally areas above 66 has been masked out (altimetry-domain). Third and fourth column: Same as second column, but with a bathymetry mask of 200 m and 50 (default mask) and 300 km coastal mask respectively.*

**Dependence on different ocean mask [mm y^{-1}]**	Full ocean*	alt-domain*	dist-coast 50 km**	dist-coast 300 km**
Greenland	0.80	0.85	**0.85**	0.87
Antarctic	0.40	0.43	**0.43**	0.43
Glaciers	0.64	0.67	**0.67**	0.68
LWS Human	0.35	0.35	**0.35**	0.35
LWS natural	0.01	0.01	**0.01**	0.01
OMrecon (Barystatic – atmosphere)	2.11	2.21	**2.23**	2.26
GRACE	2.10	2.08	**2.11**	2.10
Altimery-Steric - GIA - OBD	N/A	2.33	**2.28**	2.23

*Except areas surrounding Sumatra and Japan

**200 m bathymetry mask applied and the altimetry-domain mask
